# Machine learning approach for the prediction of 30-day mortality in patients with sepsis-associated encephalopathy

**DOI:** 10.1186/s12874-022-01664-z

**Published:** 2022-07-04

**Authors:** Liwei Peng, Chi Peng, Fan Yang, Jian Wang, Wei Zuo, Chao Cheng, Zilong Mao, Zhichao Jin, Weixin Li

**Affiliations:** 1 Department of Neurosurgery, Tangdu Hospital, Fourth Military Medical University, No.1 Xinsi Road, Xi’an, 710038 China; 2 Department of Health Statistics, Second Military Medical University, No. 800 Xiangyin Road, Shanghai, 200433 China; 3 Institute of Pathology and Southwest Cancer Center, Southwest Hospital, Third Military Medical University (Army Medical University), Chongqing, 400038 China

**Keywords:** Machine learning, Model interpretation, Sepsis-associated encephalopathy, SAE, Web-based calculator

## Abstract

**Objective:**

Our study aimed to identify predictors as well as develop machine learning (ML) models to predict the risk of 30-day mortality in patients with sepsis-associated encephalopathy (SAE).

**Materials and methods:**

ML models were developed and validated based on a public database named Medical Information Mart for Intensive Care (MIMIC)-IV. Models were compared by the area under the curve (AUC), accuracy, sensitivity, specificity, positive and negative predictive values, and Hosmer–Lemeshow good of fit test.

**Results:**

Of 6994 patients in MIMIC-IV included in the final cohort, a total of 1232 (17.62%) patients died following SAE. Recursive feature elimination (RFE) selected 15 variables, including acute physiology score III (APSIII), Glasgow coma score (GCS), sepsis related organ failure assessment (SOFA), Charlson comorbidity index (CCI), red blood cell volume distribution width (RDW), blood urea nitrogen (BUN), age, respiratory rate, PaO_2_, temperature, lactate, creatinine (CRE), malignant cancer, metastatic solid tumor, and platelet (PLT). The validation cohort demonstrated all ML approaches had higher discriminative ability compared with the bagged trees (BT) model, although the difference was not statistically significant. Furthermore, in terms of the calibration performance, the artificial neural network (NNET), logistic regression (LR), and adapting boosting (Ada) models had a good calibration—namely, a high accuracy of prediction, with *P*-values of 0.831, 0.119, and 0.129, respectively.

**Conclusions:**

The ML models, as demonstrated by our study, can be used to evaluate the prognosis of SAE patients in the intensive care unit (ICU). Online calculator could facilitate the sharing of predictive models.

**Supplementary Information:**

The online version contains supplementary material available at 10.1186/s12874-022-01664-z.

## Introduction

Sepsis-associated encephalopathy (SAE) is characterized by diffuse cerebral dysfunction resulted from a dysregulated host response without central nervous system (CNS) infection [1]. It develops in 8–70% of septic patients, based on the sepsis severity, patients’ profile, and SAE diagnostic criteria [2–5]. Symptoms in the acute stage contain sickness behavior, delirium, coma and so on. Further, survivors of the acute stage have a tendency to develop persistent neurocognitive impairment, including cognitive alterations, and even overt dementia [6–8]. It is reported that SAE was associated with longer duration of mechanical ventilation (MV) and prolonged lengths of stay (LOS) in the intensive care unit (ICU) as well as poor overall prognosis [9, 10].

Also, it was related to higher severity of scoring systems, including the Glasgow coma score (GCS), sequential organ failure assessment score (SOFA), and the Acute Physiology and Chronic Health Evaluation (APACHE II) [7, 10]. Moreover, with a mortality rate of up to 63% [3], SAE can be detrimental to patients’ health as well as add a heavy burden to the financial system. Accordingly, early identification, especially individual and measurable prediction models, and prompt management are of vital importance for the survival and prognosis of SAE patients [11]. Recently, the advent of machine learning (ML) algorithms has enabled us to predict disease events dynamically based on complicated clinical information. ML, an artificial intelligence method, can develop models “learning” from existing data [12]. Moreover, without particular model assumptions, ML, may be adept at handling intricate interactions between variables of one sort or another [13]. The present study aimed to investigate independent factors and then develop predictive models to quantitatively predict the likelihood of 30-day mortality in patients with SAE.

## Methods

### Data source

This retrospective study was conducted on the Medical Information Mart for Intensive Care (MIMIC)-IV version 1.0 [14]. Specifically, the MIMIC-IV database contained comprehensive, de-identified data of patients who have been admitted to the ICUs at the Beth Israel Deaconess Medical Center in Boston, Massachusetts, between 2008 and 2019, containing data from 383,220 admissions (single center). One author (CP) has obtained access to both databases and was responsible for data extraction (Certification number: 41657645). This study was approved by the Institutional Review Boards of Beth Israel Deaconess Medical Center (Boston, MA). Requirement for individual patient consent was waived due to the fact that all protected health information was deidentified.

### Participant selection

Inclusion criteria were patients with a diagnosis of sepsis in accordance with the Third International Consensus Definitions for Sepsis (Sepsis-3) [15]. People with an age of younger than 16 years old, ICU stays less than 48 h, primary brain injury (traumatic brain injury, ischemic stroke, hemorrhagic stroke, epilepsy, or intracranial infection), pre-existing liver or kidney failure affecting consciousness, chronic alcohol or drug abuse, and severe electrolyte imbalances were excluded from the study. In addition, for patients with multiple ICU admissions, only data of the first ICU admission of the first hospitalization were included in the analysis.

### Predictors of 30-day mortality in SAE patients

In this study, the data extracted from MIMIC-IV included age, gender, race, and coexisting disorders. Hereafter, the Charlson comorbidity index (CCI) was calculated from its component variables [myocardial infarction, congestive heart failure, peripheral vascular disease, cerebrovascular disease, dementia, chronic pulmonary disease, rheumatic disease, peptic ulcer disease, diabetes, paraplegia, renal disease, malignant cancer, severe liver disease, metastatic solid tumor and acquired immunodeficiency syndrome (AIDS)]. Additionally, we retrospectively extracted the following data: vital signs, laboratory findings, injury types, different therapy strategies and scoring systems on the first day of ICU admission. Since values were missing at random, we used multiple imputation to deal with missing data. Details of missing data are shown in Supplementary Table 1 (Table S1).

### Statistical analysis

Values were presented as means with standard deviations (if normal) or medians with interquartile ranges (IQR) (if non-normal) for continuous variables, and total numbers (%) for categorical variables. Continuous variables were compared by the t test or Wilcoxon rank sum test while proportions were compared using χ^2^ test or Fisher exact tests, if appropriate.

**Fig. 1:**
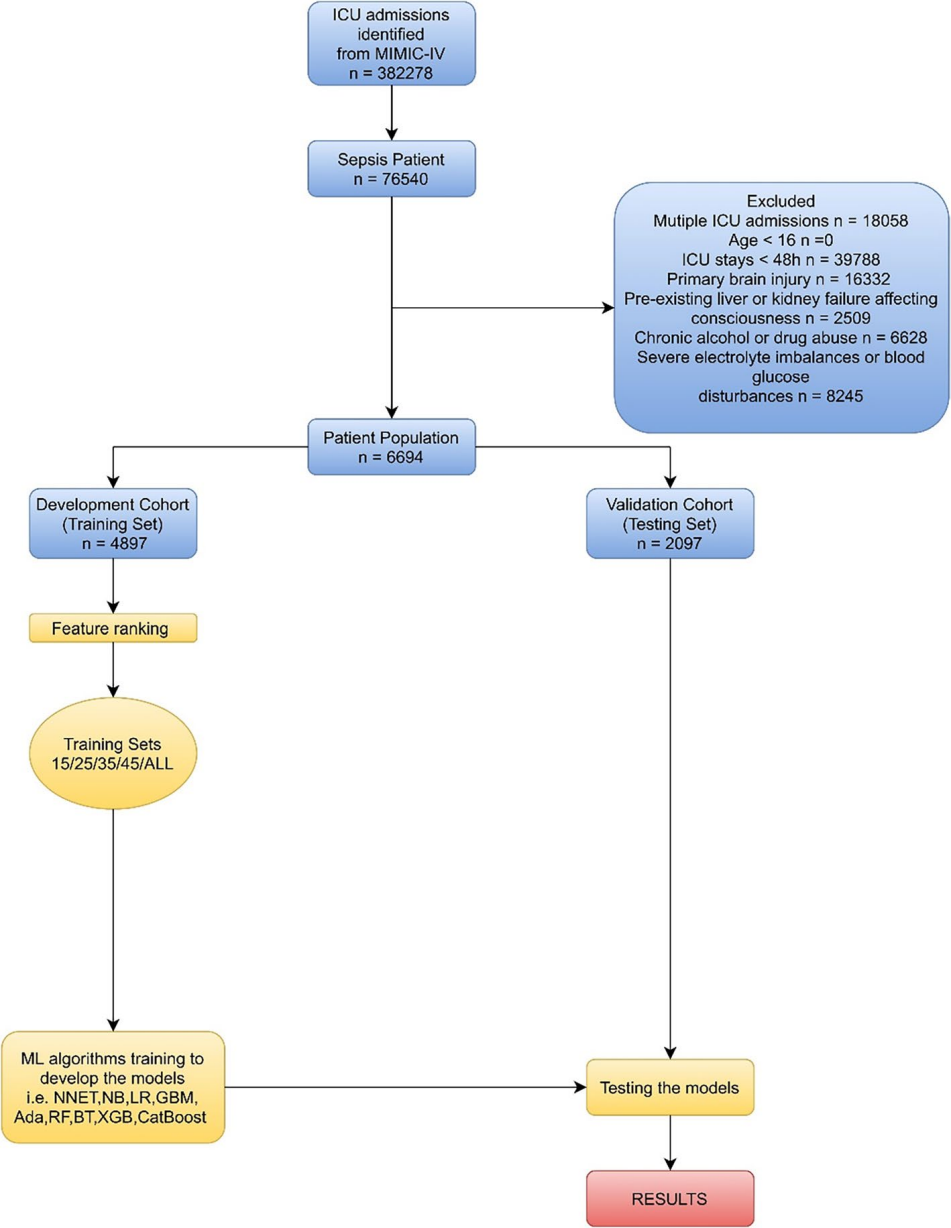
Overview of the methods used for data extraction, training, and testing. ICU, intensive care unit; MIMIC, Medical Information Mart for Intensive Care; ML, machine learning; NNET, artificial neural network; NB, naïve bayes; LR, logistic regression; GBM, gradient boosting machine; Ada, adapting boosting; RF, random forest; BT, bagged trees; XGB, eXtreme Gradient Boosting

Recursive feature elimination (RFE), a resource selection method, was utilized to select the most relevant variables. In a word, RFE recursively fits into a model based on smaller resource sets until a specified termination criterion is reached. In each loop, characteristics are classified in accordance with their importance in the trained model. Ultimately, highly correlated and collinear variables were eliminated. The characteristics were then considered in groups of 15/25/35/45/ALL (ALL = 56 variables, as represented in Fig. 1) organized by the ranks obtained after the method of selection of the characteristics. In order to find the optimal hyperparameters, fivefold cross-validation was used as the resampling method. In each iteration, every four folds were used as a training subset, and the remaining one-fold was processed to adjust the hyperparameters. This training-test process was repeated thirty times. Thus, each sample would be involved in both the training model and the testing model, so that all data were used as much as possible.

In this study, we employed nine different ML algorithms to develop models, including artificial neural network (NNET), bayes naive (NB), logistic regression (LR), gradient boosting machine (GBM), adaptating boosting (Ada), random forest (RF), bagged trees (BT), eXtreme Gradient Boosting (XGB) and CatBoost. Firstly, the population was divided into development set and validation set. As for internal validation, bootstrap resampling technique with 100 iterations was employed. Median and 95% confidence intervals of area under the curve (AUC) were calculated. Other evaluation indicators, such as, accuracy, sensitivity, specificity, negative predictive value and positive predictive value were also calculated. Moreover, the calibration curve was employed by the Hosmer–Lemeshow test of good adaptation. More precisely, the chi-square value was calculated based on the actual observed and predicted value of the model for each group and, subsequently, the corresponding *p* value was obtained. Ultimately, the “Shiny” package in R was used to build a visual data analysis platform. All analyses were performed by the statistical software packages R version 4.0.2 (http://www.R-project.org, The R Foundation). In our study, we also used the “Caret” R packages and “Shiny” R packages to achieve the process. *P* values less than 0.05 (two-sided test) were considered as statistically significant.

## Results

### Baseline characteristic

In accordance with the inclusion and exclusion criteria, 6994 patients were finally included in the dataset. The process of data extraction, training preparation, data testing by diverse ML algorithms is demonstrated in Fig. 1. The characteristics of the participants are depicted in Table 1. People who died were more likely to be older, with more comorbidities (myocardial infarction, congestive heart failure, chronic pulmonary disease, rheumatic disease, mild liver disease, renal disease, malignant cancer, severe liver disease, metastatic solid tumor), higher heart rate, higher respiratory rate, higher white blood cell (WBC), higher mean corpuscular volume (MCV), higher red blood cell volume distribution width (RDW), longer activated partial thromboplastin time (APTT), longer prothrombin time (PT), higher international normalized ratio (INR), higher lactate, higher buffer excess (BE), higher anion gap, higher potassium, higher creatinine (CRE), higher blood urea nitrogen (BUN), higher vasopressor, higher sepsis related organ failure assessment (SOFA), higher acute physiology score III (APSIII), and higher systemic inflammatory response syndrome (SIRS). Furthermore, they were more likely to have lower temperature, lower mean artery pressure (MAP), lower red blood cell (RBC), lower mean corpuscular hemoglobin concentration (MCHC), lower platelet (PLT), lower hematocrit (HCT), lower pH, lower bicarbonate, lower PaO_2_, lower chloride, lower sodium, and lower Glasgow coma score (GCS).

**Table 1:** Baseline characteristic of the MIMIC-IV cohorts

Variables	Survival (*n* = 5762)	Death (*n* = 1232)	*P* Value
**Demographics**			
Age (y), median [Q1, Q3]	70.00 (58.00,81.00)	77.00 (66.00,85.25)	< 0.001
Male, n (%)	3181 (55.21)	659 (53.49)	0.286
Race, n (%)			0.001
Black	490 (8.50)	93 (7.55)	
White	4000 (69.42)	824 (66.88)	
Hispanic	174 (3.02)	31 (2.52)	
Asian	193 (3.35)	31 (2.52)	
Others	905 (15.71)	253 (20.54)	
**Coexisting disorders, n (%)**			
Myocardial infarction	1006 (17.46)	265 (21.51)	0.001
Congestive heart failure	1977 (34.31)	540 (43.83)	< 0.001
Peripheral vascular disease	696 (12.08)	174 (14.12)	0.054
Cerebrovascular disease	259 (4.49)	51 (4.14)	0.636
Dementia	300 (5.21)	81 (6.57)	0.064
Chronic pulmonary disease	1728 (29.99)	421 (34.17)	0.004
Rheumatic disease	234 (4.06)	79 (6.41)	< 0.001
Peptic ulcer disease	229 (3.97)	64 (5.19)	0.063
Mild liver disease	709 (12.30)	242 (19.64)	< 0.001
Diabetes without complication	1308 (22.70)	255 (20.70)	0.135
Diabetes with complication	507 (8.80)	105 (8.52)	0.798
Paraplegia	124 (2.15)	17 (1.38)	0.101
Renal disease	1349 (23.41)	387 (31.41)	< 0.001
Malignant cancer	839 (14.56)	342 (27.76)	< 0.001
Severe liver disease	225 (3.90)	93 (7.55)	< 0.001
Metastatic solid tumor	343 (5.95)	205 (16.64)	< 0.001
AIDS	41 (0.71)	10 (0.81)	0.849
CCI, median [Q1, Q3]	6.00 (4.00,8.00)	7.00 (6.00,9.00)	< 0.001
**Vital signs (1st 24 h)**			
Temperature (°C), median [Q1, Q3]	36.90 (36.60,37.30)	36.70 (36.40,37.10)	< 0.001
MAP (mmHg), median [Q1, Q3]	75.00 (70.00,82.00)	73.00 (68.00,80.00)	< 0.001
Heart rate (min), median [Q1, Q3]	88.00 (77.00,100.00)	91.00 (80.00,104.00)	< 0.001
Respiratory rate (min), median [Q1, Q3]	20.00 (17.00,23.00)	22.00 (19.00,25.00)	< 0.001
**Laboratory findings (1st 24 h)**			
RBC (× 10^9^/L), median [Q1, Q3]	3.41 (3.02,3.92)	3.28 (2.88,3.80)	< 0.001
WBC (× 10^9^/L), median [Q1, Q3]	11.80 (8.60,16.03)	12.62 (8.77,17.50)	0.002
MCH (pg), median [Q1, Q3]	30.20 (28.73,31.50)	30.12 (28.70,31.63)	0.925
MCHC (%), median [Q1, Q3]	33.00 (31.85,34.00)	32.40 (31.30,33.50)	< 0.001
MCV (fL), median [Q1, Q3]	91.00 (87.00,95.00)	92.75 (88.00,97.33)	< 0.001
PLT (× 10^9^/L), median [Q1, Q3]	197.00 (139.00,268.50)	189.33 (116.71,272.75)	0.001
RDW (%), median [Q1, Q3]	14.77 (13.73,16.27)	16.00 (14.58,18.00)	< 0.001
HCT (%), median [Q1, Q3]	31.30 (27.78,35.70)	30.38 (26.80,35.07)	< 0.001
APTT (s), median [Q1, Q3]	31.90 (27.80,39.80)	35.70 (29.30,49.50)	< 0.001
PT (s), median [Q1, Q3]	14.27 (12.85,16.50)	15.70 (13.40,20.00)	< 0.001
INR, median [Q1, Q3]	1.30 (1.15,1.50)	1.40 (1.20,1.85)	< 0.001
pH, median [Q1, Q3]	7.37 (7.32,7.42)	7.36 (7.31,7.42)	< 0.001
Bicarbonate (mmol/L), median [Q1, Q3]	23.00 (20.50,26.00)	21.90 (19.00,25.50)	< 0.001
Lactate (mmol/L), median [Q1, Q3]	1.65 (1.20,2.28)	1.95 (1.40,2.89)	< 0.001
BE (mEq/L), median [Q1, Q3]	-0.50 (-3.50,1.43)	-1.33 (-5.00,1.00)	< 0.001
Aniongap (mmol/L), median [Q1, Q3]	14.00 (12.00,16.50)	15.50 (13.00,18.21)	< 0.001
PaO_2_ (mmHg), median [Q1, Q3]	112.00 (76.00,173.00)	92.00 (68.00,131.00)	< 0.001
PaCO_2_ (mmHg), median [Q1, Q3]	41.00 (37.00,47.00)	41.00 (35.00,48.00)	0.014
Chloride (mmol/L), median [Q1, Q3]	104.50 (100.50,108.20)	103.00 (98.75,107.50)	< 0.001
Calcium (mmol/L), median [Q1, Q3]	8.20 (7.73,8.63)	8.10 (7.63,8.65)	0.097
Sodium (mmol/L), median [Q1, Q3]	138.60 (136.00,141.00)	138.00 (134.82,141.43)	0.001
Potassium (mmol/L), median [Q1, Q3]	4.15 (3.83,4.55)	4.28 (3.88,4.78)	< 0.001
Glucose (mmol/L), median [Q1, Q3]	128.50 (108.33,156.24)	131.00 (107.00,163.54)	0.130
CRE (mg/dL), median [Q1, Q3]	1.05 (0.75,1.68)	1.35 (0.85,2.30)	< 0.001
BUN (mg/dL), median [Q1, Q3]	22.29 (15.00,37.67)	32.58 (20.67,52.35)	< 0.001
**Therapy (1st 24 h), n (%)**			
Vasopressor	1799 (31.22)	538 (43.67)	< 0.001
**Scoring system**			
GCS	13.00 (9.00,14.00)	8.00 (3.00,12.00)	< 0.001
SOFA	6.00 (4.00,9.00)	9.00 (6.00,12.00)	< 0.001
APSIII	55.00 (41.00,72.00)	80.50 (63.00,102.00)	< 0.001
SIRS	3.00 (2.00,3.00)	3.00 (2.75,4.00)	< 0.001

*AIDS* Acquired Immunodeficiency Syndrome, *CCI* Charlson Comorbidity Index, *MAP* Mean Artery Pressure, *RBC* Red Blood Cell, *WBC* White Blood Cell, *MCH* Mean Corpuscular Hemoglobin, *MCHC* Mean Corpuscular Hemoglobin Concentration, *MCV* Mean Corpuscular Volume, *PLT* Platelet, *RDW* Red blood cell volume Distribution Width, *HCT* Hematocrit, *APTT* Activated Partial Thromboplastin Time, *PT* Prothrombin Time, *INR* International Normalized Ratio, *pH* potential of Hydrogen, *BE* Buffer Excess, *CRE* Creatinine, *BUN* Blood Urea Nitrogen, *GCS* Glasgow Coma Score, *SOFA* Sepsis related Organ Failure Assessment, *APSIII* Acute Physiology Score III, *SIRS* Systemic Inflammatory Response Syndrome

### Variable importance

**Fig. 2:**
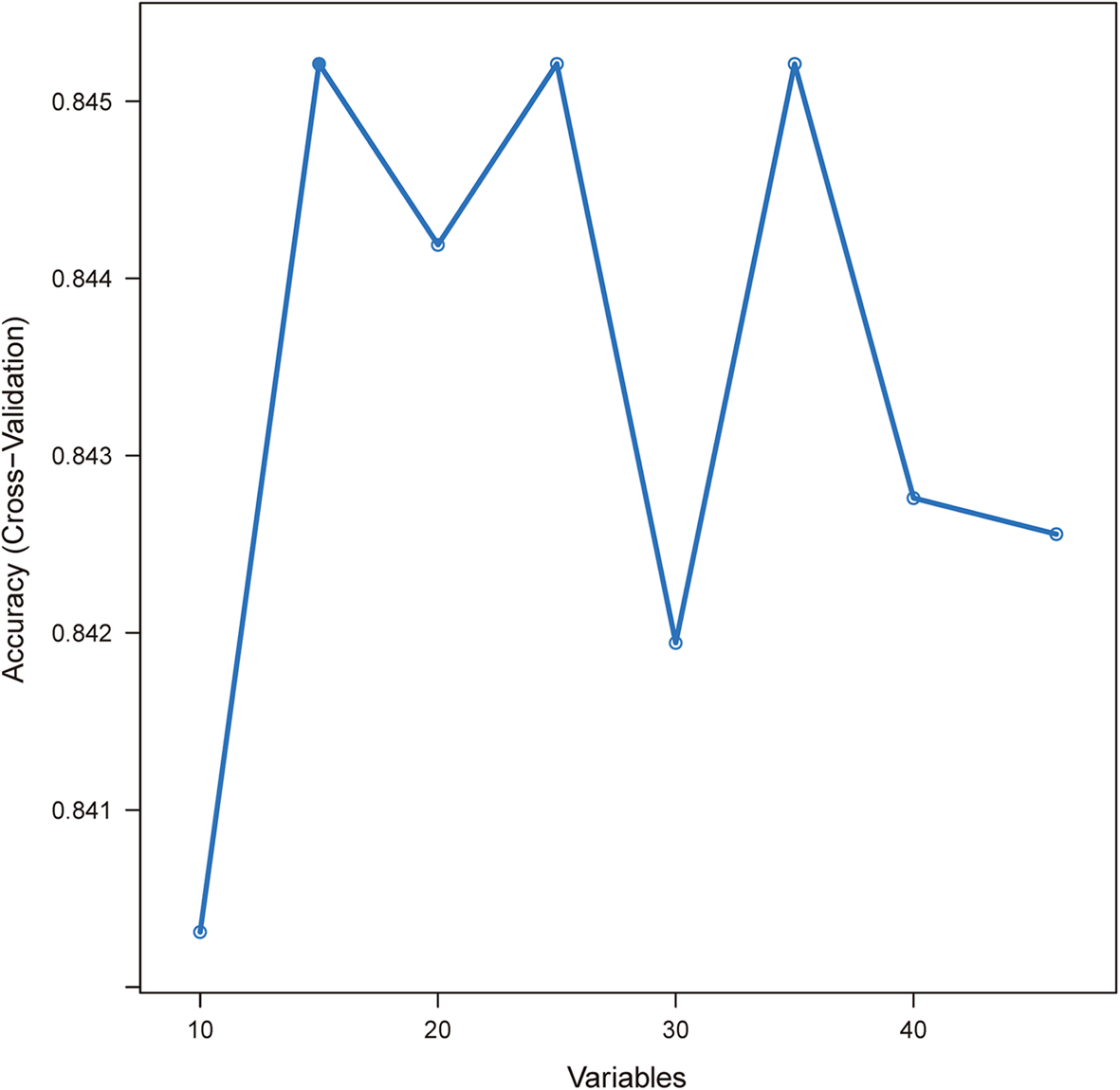
Association between the number of variables allowed to be considered at each split and the prediction accuracy in the REF algorithm. REF, recursive feature elimination

Based on the threshold measure of importance, a total of 15 important predictors were selected by the RFE algorithm. (Fig. 2) These variables included APSIII, GCS, SOFA, CCI, RDW, BUN, age, respiratory rate, PaO_2_, temperature, lactate, CRE, malignant cancer, metastatic solid tumor, and PLT. Then, these 15 variables were used in all the subsequent analysis for all models in both training and testing sets.

### Comparisons among different ML models

**Fig. 3:**
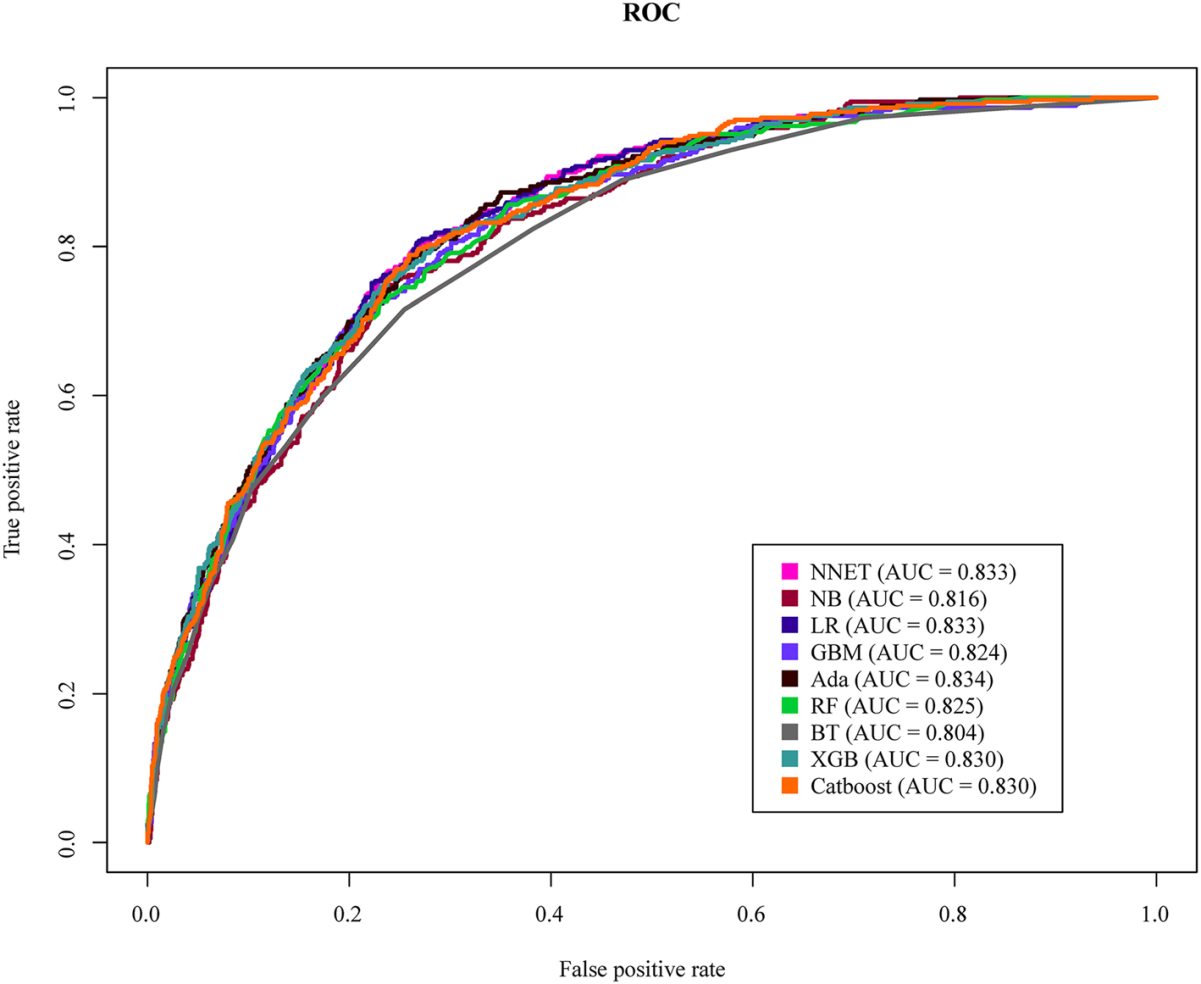
AUC of ROC curve by ML models in the validation cohort. AUC, area under the curve; ROC, receiver operate characteristics; ML, machine learning; NNET, artificial neural network; NB, naïve bayes; LR, logistic regression; GBM, gradient boosting machine; Ada, adapting boosting; RF, random forest; BT, bagged trees; XGB, eXtreme Gradient Boosting

**Fig. 4:**
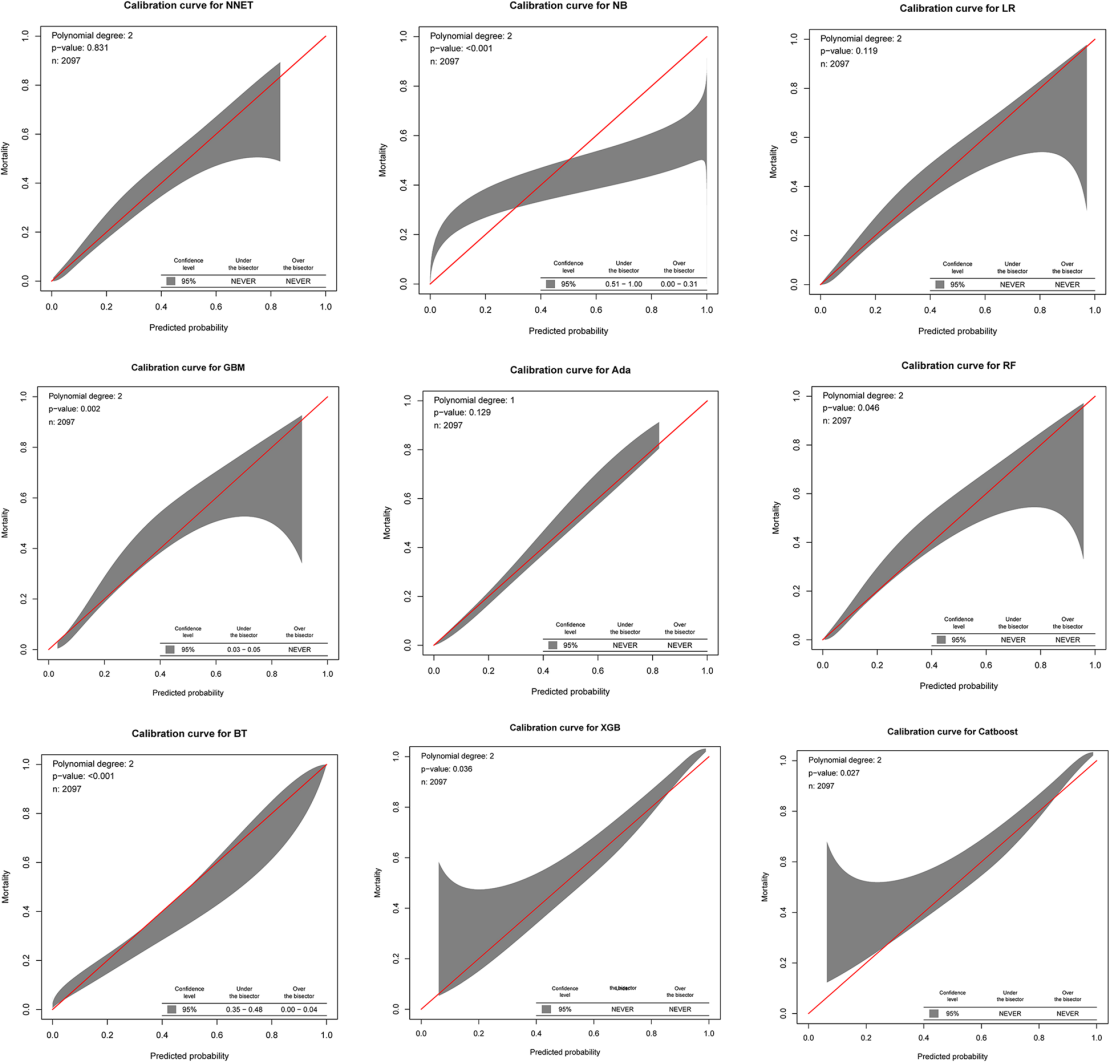
Calibration curve in the validation cohort. NNET, artificial neural network; NB, naive bayes; LR, logistic regression; GBM, gradient boosting machine; Ada, adapting boosting; RF, random forest; BT, bagged trees; XGB, eXtreme Gradient Boosting

The discriminatory abilities of all models for the prediction of 30-day mortality in SAE patients are shown in Fig. 3 and Table 2. Within the training set, the NNET, NB, LR, GBM, Ada, RF, BT, XGB, and CatBoost models were established, and the testing set obtained AUCs of 0.833, 0.816, 0.833, 0.824, 0.834, 0.825, 0.804, 0.830, and 0.830, respectively. Comparatively, the BT had the lowest discriminative ability (AUC 0.804, 95% CI 0.786 to 0.820) while the other eight models had a relatively higher discriminative ability (Table S2). In terms of the calibration performance, the NNET, LR, Ada models had a good calibration—namely, a high accuracy of prediction, with *P*-values of 0.831, 0.119, and 0.129, respectively (Fig. 4).

**Table 2:** Analysis of sensitivity and specificity

Model	Accuracy	Sensitivity	Specificity	PPV	NPV	AUC	Operating threshold	95% CI
NNET	0.840	0.802	0.733	0.391	0.946	0.833	0.164	(0.816, 0.849)
NB	0.833	0.767	0.800	0.450	0.941	0.816	0.058	(0.799, 0.833)
LR	0.843	0.808	0.731	0.391	0.947	0.833	0.162	(0.816, 0.848)
GBM	0.844	0.805	0.699	0.360	0.944	0.824	0.141	(0.807, 0.840)
Ada	0.846	0.786	0.737	0.390	0.942	0.834	0.148	(0.817, 0.849)
RF	0.840	0.856	0.642	0.338	0.954	0.825	0.150	(0.808, 0.841)
BT	0.836	0.715	0.745	0.375	0.925	0.804	0.240	(0.786, 0.820)
XGB	0.844	0.808	0.712	0.374	0.945	0.830	0.157	(0.814, 0.846)
CatBoost	0.842	0.789	0.741	0.394	0.943	0.830	0.165	(0.813, 0.846)

*PPV* Positive Predictive Values, *NPV* Negative Predictive Values, *AUC* Area Under the Curve, *CI* Confidence Interval, *NNET* artificial Neural Network, *NB* Naïve Bayes, *LR* Logistic Regression, *GBM* Gradient Boosting Machine, *Ada* Adapting boosting, *RF* Random Forest, *BT* Bagged Trees, *XGB* eXtreme Gradient Boosting

**Fig. 5:**
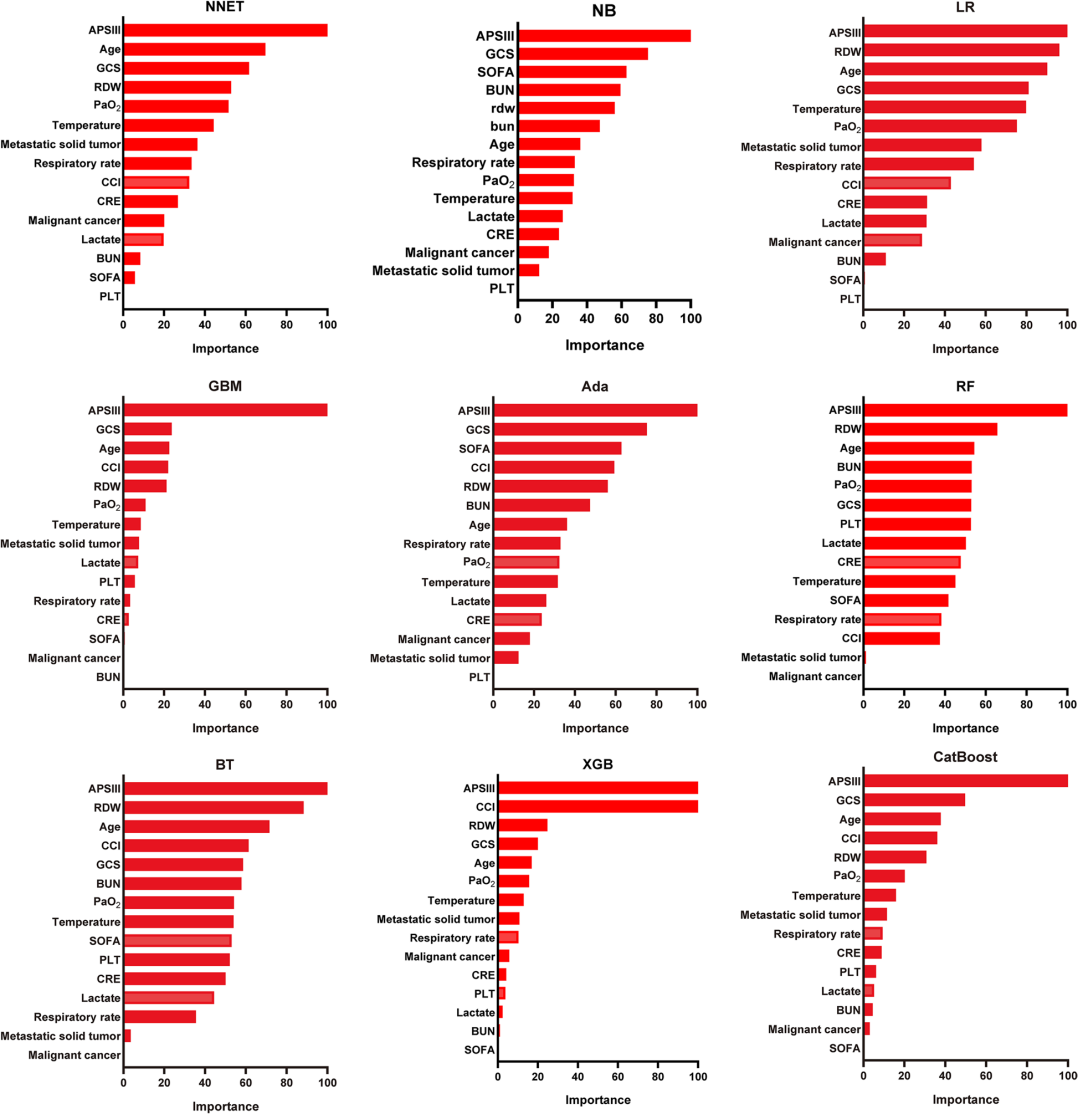
Variable importance in nine different ML models. ML, machine learning; NNET, artificial neural network; NB, naïve bayes; LR, logistic regression; GBM, gradient boosting machine; Ada, adapting boosting; RF, random forest; BT, bagged trees; XGB, eXtreme Gradient Boosting;

In the Fig. 5, fifth predictor variables in the ML are demonstrated. Each variable incorporated in the study had varying importance over SAE depending on the ML approach. In general, APSIII was the variable with greatest importance across all ML algorithms, followed by GCS, RDW, and so forth.

### Application of model

**Fig. 6:**
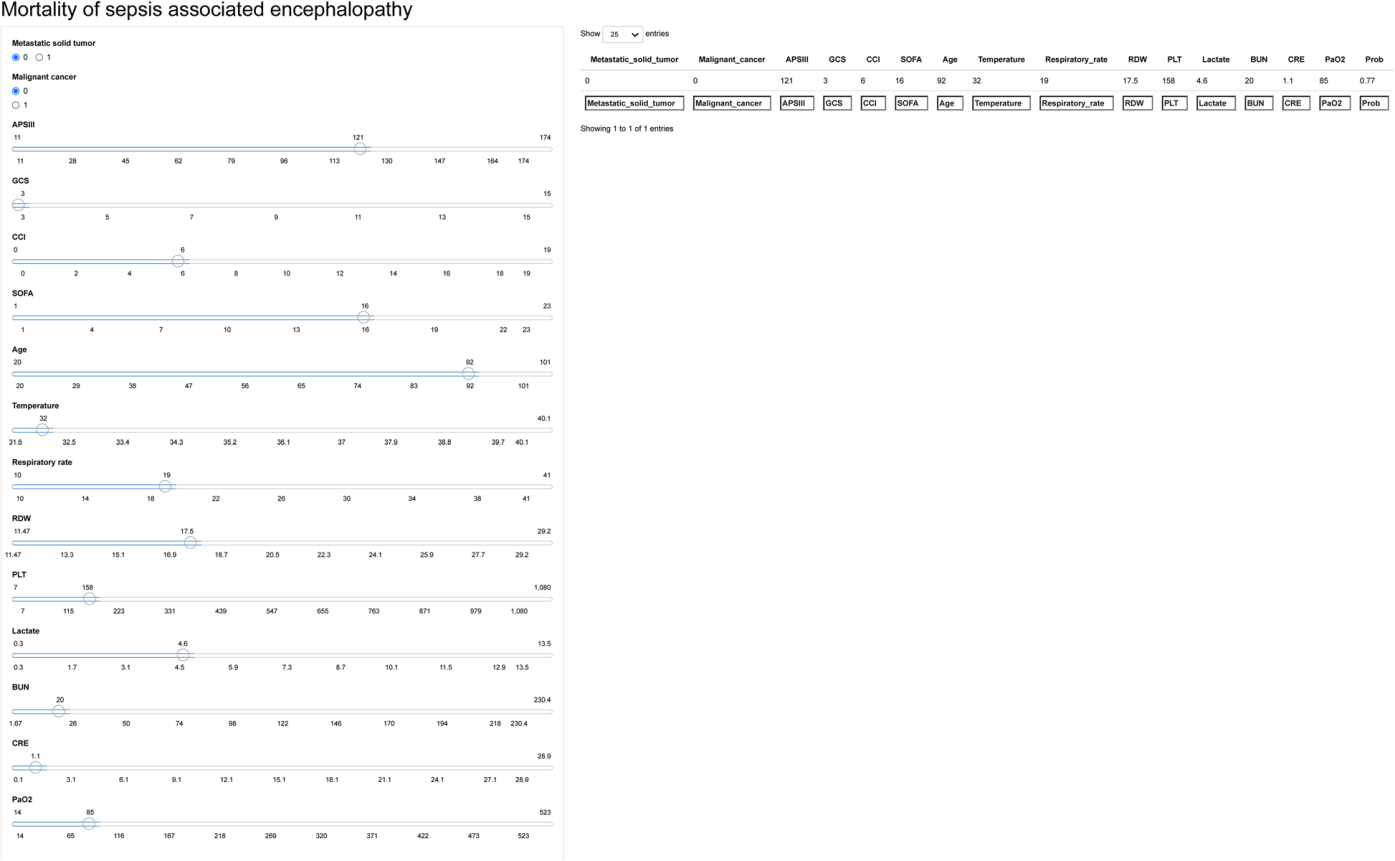
Examples of website usage. Entering the input value determined the mortality and displayed how each value contributed to the prediction. CCI, Charlson Comorbidity Index

The Shiny package analyzed the entire training set, demonstrating the impact of each variable on predicting SAE (Fig. 6). For example, the information of one patient was input into the model: no metastatic solid tumor, no malignant cancer, APSIII (121), GCS (3), CCI (6), SOFA (16), age (92), temperature (32 ℃), respiratory rate (19 per/min), RDW (17.5%), PLT (158 × 10^9^/L), lactate (4.6 mmol/L), BUN (20 mg/dL), CRE (1.1 mg/dL), PaO_2_ (85 mmHg). The model analyzed that the risk of in-hospital mortality in this patient was 84.20%, indicating that the 30-day mortality for this SAE patient was relatively high, and precaution measures were recommended. In order to better apply this model, we also made a web-based calculator (https://pengchi2009.shinyapps.io/Mortality_of_sepsis_associated_encephalopathy/).

## Discussion

Herein, nine ML models were developed and further validated to predict 30-day mortality of SAE patients. In terms of the discrimination and calibration performance, the NNET, LR and Ada model outperformed the remaining models. To make it easier for surgeons to use the model, a web-based calculator was then developed. Only by inputting the variable values can the 30-day death rate be shown. Both physicians and patients could perform an individualized prediction of the 30-day mortality of SAE, which is consistent with the personalized medicine trend. Undoubtedly, this calculator is conducive to correct clinical decisions, and more importantly, timely treatment strategy.

A study of 69 cases of sepsis patients demonstrated that in patients with no encephalopathy (*n* = 20), mild encephalopathy (*n* = 17), severe encephalopathy (*n* = 32), the mortality rate was 0, 35%, and 53%, respectively, showing that mortality was correlated to the severity of SAE [16]. In this study, fifteen variables were identified as risk factors, involving APSIII, GCS, SOFA, CCI, RDW, BUN, age, respiratory rate, PaO_2_, temperature, lactate, CRE, malignant cancer, metastatic solid tumor, and PLT.

Published study conducted by Chen J et al. [17] indicated that APACHE II and SOFA were independent risk factors for 28-day mortality in SAE patients, which was similar to our findings. A range of previous studies also have found that the mortality rate of sepsis patients is related to higher values of the GCS, SOFA, and the APACHE II score [10, 18, 19]. As an established method of summarizing patient severity of illness on admission to the ICU, APSIII is a part of the APACHE system of equations for the prediction of outcomes for ICU patients [20, 21]. In our study, APSIII and SOFA were variables with the relatively higher weight in the importance plot, demonstrating that they had strong power to predict 30-day mortality of SAE patients. It is manifest that SAE patients with multiple organ dysfunction syndrome (MODS) are associated with an increased risk of mortality. Cascade immune response, circulatory abnormalities, mitochondrial dysfunction as well as hypoxia endothelial permeability increases may be responsible for such a complicated pathophysiological process [22–25]. Consequently, the treatment of SAE is based both on the management of sepsis and on the correction of potential neurotoxic factors.

Similar to previous study conducted by Yang Y et al. [26], RDW was an important predictor for 30-day death of SAE patients. Although the mechanism remains, to a wide extent, unclear, it is estimated that inflammation reaction and oxidative stress might invite an increase in RDW values, and simultaneously, these mechanisms may play a pivotal role in the poor prognosis of SAE [27–29]. Furthermore, in keeping with previous study, we also unearthed that age was independently associated with 30-day mortality [17]. It was probably attributable to the fact that elderly patients exhibit a higher risk and mortality from sepsis [30]. Further, our research offered insight into the fact that renal function (BUN, CRE), respiratory rate, PaO_2_, and PLT were identified as predictors for 30-day death in SAE patients. Previous study also found that sepsis patients with renal or multi-organ failure were more frequently affected than those without organ complications [3].

Additionally, renal function alteration is not only associated with biological alterations including severe acidosis and uremia but also associated with neurotoxic substances accumulation, such as, antibiotics and hypnotics [10]. Notably, caused by the enhanced activation of cytokine expression and vascular endothelial cells, platelet abnormalities may proceed to disseminated intravascular coagulation (DIC) [31, 32]. Accordingly, it is advisable that we properly improve respiratory and circulation status, and correct coagulation function to reduce the mortality of SAE patients.

Result from previous study indicated that temperature and lactate were significantly correlated with mortality in SAE patients [33], these findings have also been confirmed in our research. As is well known, lactate was an important indictor which reflected the prognosis of sepsis patients [34, 35]. In other words, serum lactate was used to evaluate disease severity and guide treatment plan [34], thereby indicating that SAE patients experienced microcirculation obstacles, which may induce tissue ischemia and hypoxia. Accordingly, for patients with lactate acidosis and hyperlactic acidosis, timely rehydration and other treatments are needed.

The strengths of this study lied in the fact that it applied modern ML approaches to predict 30-day mortality, ensured that surgeons can conduct triage of patients at risk timely. Another important point to note was that the use of cross-validation is instrumental in decreasing potential overfitting. Further, based on a real-world data with relatively large samples (*n* = 6994), this study underwent a rigorous statistical test.

There were limitations in this study. First, although cohorts were divided into training set and validation set (70%: 30%), external verification is still a necessity. And compared to traditional models, the evaluation indicators including AUCs and accuracy of Ada have a slight edge. Second, as an administrative database, there exist inherent limitations. For example, the neuroimaging data were not available. Third, as with all potential retrospective studies, there was a potential for unmeasured confounders. Fourth, since it was based on ICU patients, this study could not be generalizable to other population. Lastly, although the shiny package was utilized to help visualize the results, a more applicable model is still needed in clinical practice.

## Conclusions

On a whole, ML models, are able to individually predict 30-day mortality in SAE patients. and thereby assisting in the early screening for SAE patients who are at risk. This is particularly crucial as early treatment may facilitate the neurocognitive outcome. Future studies should be concentrated on investigating the long-term prognosis of SAE patients and the underlying mechanism of SAE.

## Supplementary Information

**Additional file 1:** **Table S1.** Missing number (%) for included variables in the dataset. **Table S2.** The comparison of different ROC curve by De Long’s method. 

## Data Availability

Publicly available datasets were analyzed in this study. These data can be found in the physionet (https://physionet.org/content/mimiciv/1.0/).
