# Supplementary material for: Machine learning approach for the prediction of 30-day mortality in patients with sepsis-associated encephalopathy
Source: BMC Med Res Methodol. 2022 Jul 4;22:183. doi: 10.1186/s12874-022-01664-z (PMC9252033; doi:10.1186/s12874-022-01664-z)
Supplement: Supplementary file 1 — Additional file 1: Table S1. Missing number (%) for included variables in the dataset. Table S2. The comparison of different ROC curve by De Long’s method. [file 12874_2022_1664_MOESM1_ESM.docx]

| Table S1 Missing number (%) for included variables in the dataset | |
| --- | --- |
| Variables | Missing, (%) |
| Temperature | 0.04 |
| MAP | 0.17 |
| Heart rate | 0.17 |
| Respiratory rate | 0.20 |
| RBC | 0.56 |
| WBC | 0.17 |
| MCH | 0.57 |
| MCHC | 0.57 |
| MCV | 0.57 |
| PLT | 0.17 |
| INR | 7.26 |
| PT | 7.26 |
| APTT | 7.81 |
| RDW | 0.56 |
| HCT | 0.17 |
| pH | 16.62 |
| Bicarbonate | 0.01 |
| BE | 16.62 |
| Anion gap | 0.30 |
| PaO2 | 16.62 |
| PaCO2 | 16.62 |
| Chloride | 0.17 |
| Calcium | 4.93 |
| Sodium | 0.17 |
| Potassium | 0.17 |
| Glucose | 0.34 |
| CRE | 0.14 |
| BUN | 0.17 |
| Lactate | 18.29 |

MAP, mean artery pressure; RBC, red blood cell; WBC, white blood cell; MCH, mean corpuscular hemoglobin; MCHC, mean corpuscular hemoglobin concentration; MCV, mean corpuscular volume; PLT, platelet; INR, international normalized ratio; PT, prothrombin time; APTT, activated partial thromboplastin time; RDW, red blood cell volume distribution width; HCT, hematocrit; BE, buffer excess; CRE, creatinine; BUN, blood urea nitrogen;

Table S2 The comparison of different ROC curve by De Long’s method

| *P* value | NNET | NB | LR | GBM | RF | ada | TreeBag | XGB |
| --- | --- | --- | --- | --- | --- | --- | --- | --- |
| NB | 0.004 |  |  |  |  |  |  |  |
| LR | 0.553 | 0.005 |  |  |  |  |  |  |
| GBM | 0.051 | 0.223 | 0.057 |  |  |  |  |  |
| RF | 0.119 | 0.214 | 0.136 | 0.934 |  |  |  |  |
| Ada | 0.826 | 0.005 | 0.775 | 0.026 | 0.071 |  |  |  |
| BT | 0.001 | 0.187 | 0.001 | 0.008 | 0.001 | <0.001 |  |  |
| XGB | 0.537 | 0.023 | 0.580 | 0.068 | 0.232 | 0.362 | 0.001 |  |
| CatBoost | 0.410 | 0.017 | 0.449 | 0.057 | 0.221 | 0.297 | <0.001 | 0.836 |

NNET, artificial neural network; NB, naïve bayes; LR, Logistic regression; GBM, gradient boosting machine; Ada, adapting boosting; RF, random forest; BT, bagged trees; XGB, eXtreme Gradient Boosting
